# Merkel cell carcinoma in the left groin: A case report and review of the literature

**DOI:** 10.3892/ol.2015.2861

**Published:** 2015-01-09

**Authors:** WENTING HE, DACHUAN ZHANG, JINGTING JIANG, YANPING CHEN, CHANGPING WU

**Affiliations:** 1 Department of Oncology, The Third Affiliated Hospital of Soochow University, Changzhou, Jiangsu 213003, P.R. China; 2 Department of Pathology, The Third Affiliated Hospital of Soochow University, Changzhou, Jiangsu 213003, P.R. China; 3 Department of Geriatric Medicine, Wuxi No. 2 Hospital Affiliated to Nanjing Medical University, Wuxi, Jiangsu 214002, P.R. China

**Keywords:** Merkel cell carcinoma, neuroendocrine carcinoma, histopathology, immunohistochemistry, differential diagnosis

## Abstract

Merkel cell carcinoma (MCC) is a relatively rare but aggressive primary neuroendocrine carcinoma of the skin. MCC frequently occurs on sun-exposed areas in elderly Caucasian patients and has a propensity for local recurrence, regional lymph node invasion and distant metastases. However, MCC occurring on sites that are not sun-exposed in Asian patients is extremely uncommon. The current study describes the case of a 66-year-old Chinese male who presented with an asymptomatic, smooth lesion in the left inguinal region, which was initially diagnosed as a malignant lymphoma. Upon histological and immunohistochemical analysis, the tumor was consistent with the diagnosis of an MCC. In conclusion, due to its low incidence rate and lack of characteristic clinical manifestations, the final diagnosis of MCC relies on the analysis of histological findings and immunohistochemical markers following lesion biopsy or resection. The present study aimed to report a case of MCC and present a brief literature review in order to bring attention to the diagnosis of this condition.

## Introduction

Merkel cell carcinoma (MCC), also known as trabecular carcinoma of the skin, is a primary cutaneous neuroendocrine malignancy with a low incidence rate and a high rate of aggressive biological behavior. The tumor most commonly occurs in the sun-exposed areas of Caucasians aged >50 years; primarily on the head and neck, followed by the extremities, trunk and buttocks (1,2). The clinical manifestation of MCC is that of a solitary, firm, glossy, painless cutaneous nodule, with red- or purple-colored skin, which may exhibit ulcerative characteristics. Histologically, MCC exhibits sheets of monomorphous small blue cells, which may be confused with other closely associated skin neoplasms, such as small cell lung cancer (SCLC), cutaneous lymphoma, melanoma, Ewing’s sarcoma and rare basal cell carcinoma (3). The positive expression of certain antibodies in immunohistochemical staining is confirmed to be an important diagnostic tool to distinguish MCC from these tumors.

A retrospective review was previously conducted by Song *et al* (4) to describe the clinical profile of MCC in China. The results indicated that MCC appeared to be uncommon in mainland China, and that patients often developed lesions on the head/neck region, as observed in Western countries, but received surgery alone as treatment. The present study reports the case of a Chinese male who presented with an unusual nodule in the left groin, without sun exposure, which was initially diagnosed as a malignant lymphoma, but was later proven to be an MCC following immunohistochemical studies. Written informed consent was obtained from the patient and the patient’s family.

## Case report

A 66-year-old Chinese male presented to the Deaprtment of Pathology, The Third Affiliated Hospital of Soochow University (Changzhou, China) in May 2009 with complaints of a 2-cm asymptomatic, smooth and firm nodule in the left inguinal region. There was no discoloration or other visible abnormality of the overlying skin. Surgical excision of the lesion was performed and a diagnosis of malignant lymphoma was formed at the Changzhou No.2 People’s Hospital (Changzhou, China). The patient’s medical history revealed a previous resection of a similar painless nodule in the subcutaneous region of the left knee 6 months previously, but a pathological examination had not been performed. The nodule in the left groin was suspected to be a metastatic lesion.

**Figure 1:**
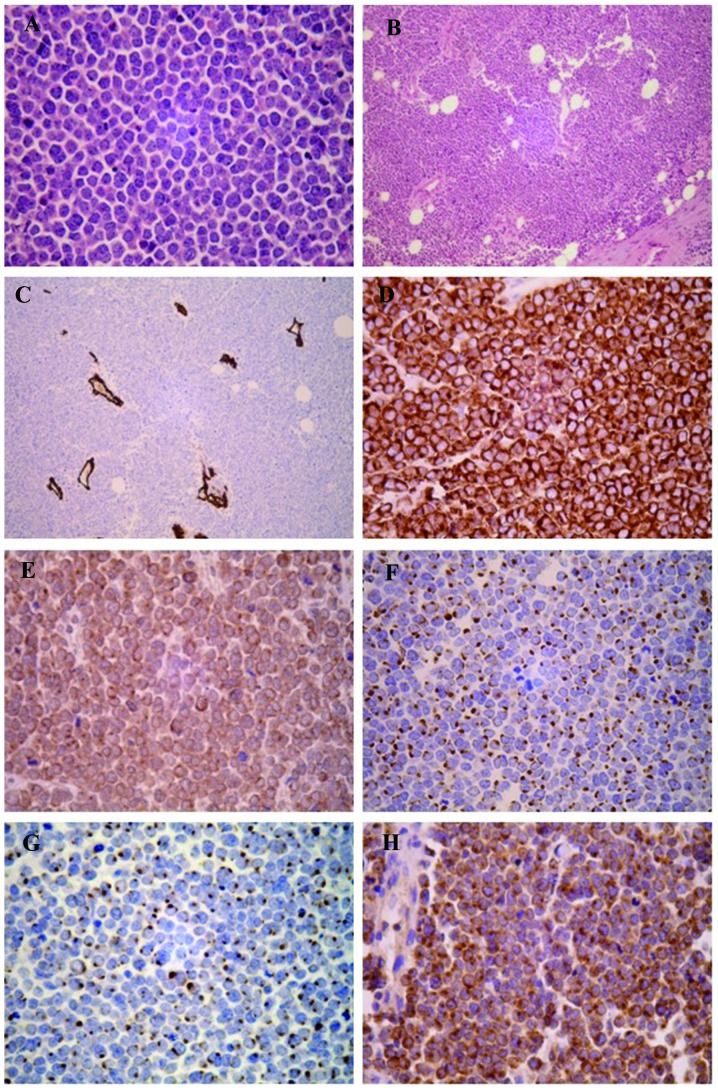
(A) Sheets of round to oval, small, blue cells with amphophilic sparse cytoplasm and vesicular nuclei [hematoxylin and eosin (HE); magnification, ×400). (B) Diffuse distribution of neoplastic cells and deep mesenchyme infiltration (HE; magnification, ×100). (C) Vascular proliferation in interstitial substance with staining for cluster of differentiation 34 (magnification, ×100). (D) Merkel cell carcinoma (MCC) with diffusely-positive staining for synaptophysin (magnification, ×400). (E) MCC with diffusely-positive staining for chromogranin (magnification, ×400). (F) Positive staining for cytokeratin (CK)20, with a perinuclear dot-like pattern, supporting the diagnosis of MCC (magnification, ×400). (G) Positive staining for CK8/18, with a perinuclear dot-like pattern (magnification, ×400). (H) MCC with diffusely-positive staining for epithelial membrane antigen (magnification, ×400).

Upon review of 4-μm sections of the lesion by light microscopy, the entire thickness of the dermis was observed to be widely infiltrated by small round monomorphic cells, with minimal cytoplasm, hyperchromatic nuclei and small nucleoli (Fig. 1A). Apparent nuclear atypia and multiple mitotic figures were observed. These neoplastic cells showed diffuse distribution and infiltrated into the deep mesenchyme, where blood vessels were plentiful (Fig. 1B and C). Immunohistochemical staining was performed on 4-μm thick, formalin-fixed, paraffin-embedded tissue sections provided by the external hospital. The results demonstrated that the tumor cells were strongly positive for neuroendocrine markers, including chromogranin A (CgA) and synaptophysin (Syn), and epithelial markers cytokeratin (CK) 20, CK8/18 and epithelial membrane antigen (EMA) (Fig. 1D–H), but negative for leukocyte common antigens (LCA), thyroid transcription factor-1 (TTF-1), Melan-A, human melanoma black 45 (HMB45), vimentin (Vim), S-100, cluster of differentiation (CD)34, CD57 and CD99. These histopathological and immunohistochemical features were consistent with a diagnosis of MCC. Therefore, a corrected diagnosis of MCC was made for this patient.

## Discussion

MCC was first described by Toker (5) in 1972 and is believed to be a rare skin carcinoma of neuroendocrine origin. Fair skin shows a clear predilection for MCC, representing nearly 95% of the total number of cases. MCC is less commonly described in the skin types of patients of Asian, Native American or African descent (6). Epidemiology and End Results (SEER) from 1973 to 2006, 94.9 % of patients were Caucasioan, African-Americans represented only 1% of patients (7). However, the histogenesis of MCC remains controversial. The most commonly accepted hypothesis is that the tumor arises from a neural crest-derived cell, which is considered to be the Merkel cells (8). However, recent observations have challenged this concept and put forward a pluripotent cutaneous stem cell origin (9). MCC is a challenging and aggressive disease, with high mortality and associations with Merkel cell polyomavirus and immunosuppression. Even after radical surgery, it easily relapses *in situ*, invades the regional lymph nodes and metastasizes to distant skin, liver, bones and lungs, and more rarely to organs such as the pancreas (3,10).

Due to the nondescript clinical features of MCC, the diagnosis in the majority of cases relies upon the pathological examination. Microscopically, MCCs frequently originate in the dermis and mostly invade the lymphatic capillaries of subcutaneous adipose tissue; <10% of cases have a tendency to spread into the epidermis and may even generate micro-abscesses (11). Histologically, the tumor is composed of small, round to oval-shaped, basophilic cells that are uniform in size, with little cytoplasm, vesicular nuclei, finely granular dispersed chromatin, distinct nuclear membranes and multiple small nucleoli. Numerous mitotic figures and apoptotic bodies are usually present. Additionally, certain MCC cases present with increased vascularity, which is significant as increased vascular proliferation is associated with a worse prognosis, as are lymphovascular invasion, a small cell size and a high mitotic rate.

According to the varying pathological morphology, MCC can be histologically divided into three subtypes: The trabecular, intermediate cell and small cell types. There does not appear to be any prognostic differences associated with these subtypes. The rare trabecular type displays uniform cells with characteristic parallel alignment and Zellballen architecture. Cytology shows vesicular nuclei and inconspicuous nucleoli. The intermediate cell variant is observed most commonly in MCC and displays a solid, diffuse growth pattern made up of closely packed cells that are shaped like lymphocytes. Mitoses and nuclear fragmentation are noted frequently in tumor cells in this particular pattern. The tumor cells of the small cell type are characterized by deeply stained ‘oat cells’ and possess obvious nuclei, scant cytoplasm, spotty necrosis and nuclear debris. Mixed and transitional forms of the three types are often present (12,13).

Upon immunohistochemical analysis, the tumor cells of MCC are labeled with neuroendocrine markers (CgA, Syn and NSE) and epithelial markers such as CK20 and CK8/18, which may show a characteristic perinuclear-dot pattern. This feature is routinely used to assist in diagnosing MCC. Indeed, a previous study recorded that 87% of 191 MCC cases were positive for CK20 (14). Thus, the lack of a characteristic stain for CK20 does not exclude the diagnosis of MCC. Several studies have also found CD117 and CD99 positivity in cases of MCC, and CD44-positive cases may correlate with the high risk of tumor metastasis (15,16). In order to form a differential diagnosis in the present study, these tests were combined with staining for LCA, Vim, TTF-1, HMB45, Malen-A and S-100, which are usually negative in the majority of MCC.

In the majority of cases, the diagnosis of MCC can be challenging due the uncharacteristic histomorphological cellular features of MCC and the extensive list of differential diagnoses. Immunohistochemical staining plays a crucial role in the differential diagnosis of these tumors. Characteristic immunohistochemical staining of MCC and other small, round, blue cell tumors is compared in Table I. Lymphoma is a critical differential diagnosis of MCC. The tumor cells of lymphoma have a diffuse growth pattern with plentiful cytoplasm, often infiltrating into the epidermis. The presence of irregular nuclear membranes is usually typical of lymphomas, whereas the nuclear contours in MCC are usually smooth and rounded. Specific expression patterns of LCA in malignant lymphoma can aid in establishing a definitive diagnosis (17). Similar to the small cell type of MCC, the tumor cells of metastatic SCLC are small with deeply stained nuclei. It should be noted that neuroendocrine markers are not specific for MCC, as they can also be positively expressed in metastatic SCLC. When the distinction is problematic, positive staining for TTF-1 and CK7 and negative staining for CK20 in metastatic SCLC offer the greatest sensitivity and specificity, however, CK20 may be positive in 3% of the SCLC, which should be taken into consideration (18). The conventional, reliable, morphological feature of tumor cells being pleomorphic and often involving the epidermis can be of aid in distinguishing non-pigmented malignant melanoma from MCC. When in doubt, the immunohistochemical stains of Malen-A, HMB45 and S-100, which are expressed in the majority of malignant melanomas, provide valuable evidence (19). In primitive neuroectodermal tumors (PNETs), characteristic rosette-like structures can be observed, and the central lumen are filled with hyperplastic fibrils. The common expression of CD99 in PNET and CK20 in MCC suggests these markers may be valuable in the diagnostic setting (20). Other small cell cutaneous carcinomas, such as primary poorly-differentiated squamous carcinoma of the skin, can also be confused with MCC in terms of the morphological features. Only epithelial markers, including CEA and EMA, can be used in the staining of the tumor cytoplasm of squamous carcinoma. Occasionally squamous carcinoma can occur concurrently with MCC, however, MCC has a poorer prognosis.

In conclusion, MCC occurring on sites not exposed to the sun, such as the inguinal region, is rare. Due to the low incidence rate and lack of characteristic clinical manifestations, MCC is often misdiagnosed. The final diagnosis relies on the analysis of histological findings and immunohistochemical markers following lesion biopsy or resection.

## Figures and Tables

**Table I:** Immunohistochemical staining markers of small, round, blue cells in the skin.

Cancer type	CK20	CEA	EMA	CgA	Syn	NSE	TTF-1	Melan-A	HMB45	S-100	CD56	CD99	LCA
Merkel cell carcinoma	+/−	−	+	+/−	+/−	+	−	−	−	−	+	−/+	−
Small cell lung cancer	−/+	+	−	+/−	+/−	+	+/−	−	−	−	+	−	−
Malignant melanoma	−	−	−	−	−	−	−	+	+	+	−/+	−/+	−
Lymphoma	−	−	−	−	−	−	−	−	−	−	−	−	+
PNET	−	−	−	+	+	+	−	−	−	+/−	−	+	−
Squamous carcinoma	−	+	+	−	−	−	−	−	−	−	−	−	−

CK, cytokeratin; CEA, carcinoembryonic antigen; EMA, epithelial membrane antigen; CgA, chromogranin A; Syn, synaptophysin; NSE, neuron-specific enolase; TTF-1, thyroid transcription factor-1; HMB45, human melanoma black 45; CD, cluster of differentiation; LCA, leukocyte common antigen; PNET, primitive neuroectodermal tumor.
